# Utilisation of maternal healthcare services and influencing factors in public health facilities in Addis Ababa, Ethiopia

**DOI:** 10.4102/hsag.v29i0.2694

**Published:** 2024-10-09

**Authors:** Sintayehu A. Temesgen, Thinavhuyo R. Netangaheni

**Affiliations:** 1 Department of Health Studies, College of Human Sciences, University of South Africa, Pretoria, South Africa; 2 Ethiopian Public Health Institute, Addis Ababa, Ethiopia; 3 The African Center for Early Childhood Development, Addis Ababa, Ethiopia

**Keywords:** antenatal care, delivery service, Addis Ababa Ethiopia, maternal health, postnatal care, public health facilities, utilisation

## Abstract

**Background:**

Ethiopian maternal mortality remains high, despite the implementation of extensive health programmes. This indicates that the full potential of maternal health services is not being effectively utilised.

**Aim:**

This study aimed to evaluate the utilisation and factors influencing maternal healthcare services in public health facilities in Addis Ababa.

**Setting:**

This study was conducted in five public hospitals and 10 public health centres of Addis Ababa city administration from 31st August 2023 to 13th October 2023.

**Methods:**

The study’s design utilised a cross-sectional quantitative technique, which involved interviewing 354 women from each group who received visits throughout their pregnancy, delivery and postpartum period. The data were analysed using SPSS version 26.

**Results:**

This study analysed maternal health service utilisation indicators, revealing a 70.8% overall utilisation of services, with antenatal care (ANC) at 85.5%, delivery at 71.58% and family planning services (PNC) at 55.4%. The study found that the length of time spent travelling to public health facilities significantly impacts the use of maternal health services. Pregnant women who travelled less than 30 min used services 2.29 times more than those over 2 h. The average client wait time also influenced service usage. Pregnant women with four or more prenatal care visits were more likely to use services.

**Conclusion:**

The study conducted in Addis Ababa’s capital city revealed that the utilisation of maternity health care services is not optimal, despite the concentrated resources.

**Contribution:**

The findings of the study could be beneficial for the Addis Ababa Health Bureau, Ministry of Health, legislators, and other stakeholders. It can help in the development of affordable intervention programmes, filling knowledge gaps and updating scientific understanding.

## Introduction

### Utilisation of maternal health services

Ethiopia’s public healthcare system is organised into three tiers: The primary healthcare unit (PHCU) is the first tier. This is the district health system, or level one, which consists of a primary hospital and health centres along with satellite health posts. Health extension workers (HEWs) are responsible for the staffing and management of health posts, where they provide a specific and limited range of services. General hospitals are categorised as level two, representing secondary healthcare units. On the other hand, comprehensive referral hospitals are classified as level three, representing tertiary healthcare units (FMoH 2014). Every woman in Ethiopia has free access to maternity care at all three levels of the healthcare system (FMoH 2021). Blood transfusions and caesarean sections are among the complementary procedures offered (FMoH 2021).

**FIGURE 1:**
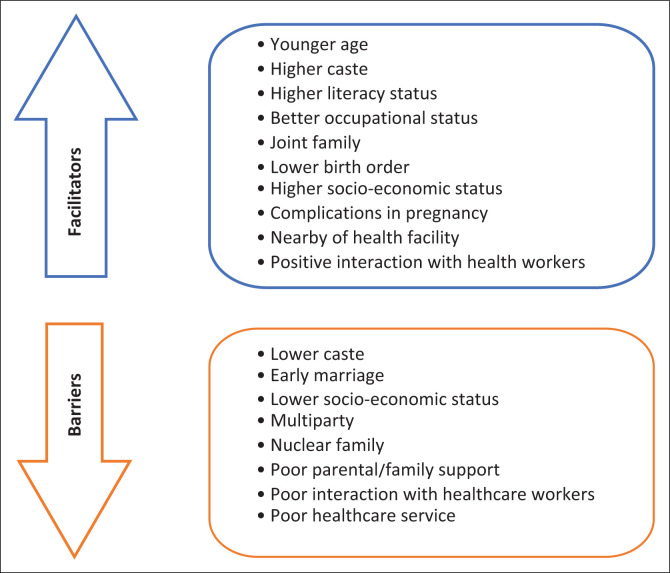
Schematic presentation of factors that determine maternal healthcare service utilisation.

A holistic approach to care during pregnancy, childbirth and postnatal periods is crucial for improving maternal health and preventing maternal mortality (Bhandari, Sarma & Kutty 2015). Maternal health service utilisation is a significant public health challenge, particularly among marginalised populations (Nuryana, Viwattanakulvanid & Romadlona 2022; Zhao et al. 2020). Antenatal coverage, institutional delivery and postnatal coverage should be considered to assess health services utilisation (Kumar & Reshmi 2022). Factors such as women’s characteristics, environmental situations and healthcare system accessibility contribute to the use of maternal health care (Erickson et al. 2021; Mweemba et al. 2021; WHO 2016). Figure 1 shows the determinant factors that affect the utilisation of maternal healthcare services.

In Ethiopia, the high rates of maternal mortality and morbidity are linked with inadequate access, poor quality and low utilisation rate of healthcare services (Tarekegn, Lieberman & Giedraitis 2014:2). Limited information exists regarding the utilisation of maternal healthcare services, despite the fact that doing so can offer chances for the early detection of health issues affecting mothers (WHO 2019:5, 2021:1).

Ethiopia has one of the highest maternal and infant mortality rates globally and this is largely because of the low utilisation of modern healthcare services by a significant proportion of women. Studies show that only 25% of Ethiopian women receive antenatal care and less than 10% receive professionally assisted delivery care. In Ethiopia, the number of pregnant women who did not receive antenatal care from a qualified practitioner was 76.4% (Seboka, Mamo & Mekonnen 2022). Despite the fact that maternal healthcare utilisation is essential for further improvement of maternal and child health, little is known about the current magnitude of use and factors influencing the use of these services in Addis Ababa. This study aimed to assess the utilisation of maternal healthcare services in Addis Ababa and identify factors influencing their use, focussing on women attending ANC, giving birth and visiting postnatal care services (PNC).

## Research methods and design

### Study design

This observational analytical study design involving cross-sectional sampling was carried out at the health facility level to collect quantitative data from the target health facilities and study participants.

### Setting

This study was conducted across the 11 sub-cities of Addis Ababa, the capital city of Ethiopia from 31st August 2023 to 13th October 2023. The city has an estimated area of 526.99 square kilometres, an estimated 3 854 863 total population (consisting of 1 389 817 male and 1 527 478 female) and an estimated density of 5535.8 people per square kilometre. Administratively, the city is subdivided into 11 sub-cities and 120 districts. Almost 100% of the population were urban dwellers (CSA 2017:34). There are 13 public hospitals (of which 7 are under the Federal Government), 97 health centres, 42 private hospitals, 1389 private different-level clinics (primary, medium, and speciality clinics and speciality centres (FMoH 2023:21).

### Study population and sampling strategy

The survey included respondents from five public hospitals and a representative sample of 10 health centres using probability sampling of simple random sampling. To randomly select the facilities, the lottery method was used whereby two boxes containing: (1) cards with names of public hospitals and (2) cards with names of health centres were used for drawing names of places. The researcher randomly picked five cards from the hospital boxes and 10 cards from the health centre boxes, thus ensuring each facility has an equal chance of selection. The reason for the selection of these health facilities was to ensure adequate representation of the quantitative samples from the hospitals and health centres.

For the exit interview, the largest and equal number of 354 mothers sample size was taken from each group of women who attended ANC, birth and PNC, even though separate samples were calculated for each attendance group. The sample size was computed using the prevalence (proportion) formula. To enhance the reliability of the sample and to capture enhanced odds of outliers, this study took the largest sample (see Equation 1 and Equation 2):

$$\text{n }(\text{ANC}/\text{Delivery}/\text{PNC})=\frac{{\text{Z}}_{\alpha /2}(\text{P}(1-\text{P})}{{\text{d}}^{2}}$$ [Eqn 1]

$$\text{n }(\text{ANC}/\text{Delivery}/\text{PNC})=\frac{{\left(1.96\right)}^{2}(0.7)(0.3)}{{\left(0.05\right)}^{2}}=323$$ [Eqn 2]

By adding a 10% non-response rate (32), the sample size will become 354 mothers on ANC.

Where *P* is service utilisation at the National level of Ethiopia (70% for antenatal care [ANC] [FMoH 2021a:21], 26.2% for delivery [Ketemaw et al. 2020] and *P* = 85% [FMoH 2021a:31]). Non-response rate of 10% was added for each initial sample.

The survey involved respondents from 5 public hospitals and 10 health centres using probability sampling of simple random sampling. The lottery method was used to randomly select facilities, ensuring equal representation of samples. To randomly select the facilities the lottery method was used whereby two boxes containing: (1) cards with names of public hospitals and (2) cards with names of health centres will be used for drawing names of places. The researcher will randomly pick five cards from the hospital boxes and 10 cards from the health facilities boxes, thus ensuring an equal chance of selection and adequate representation of the samples from the health facilities. The study recruited participants from public health facilities in each sub-city, based on the total number of maternal healthcare service users within 3 months. Systematic random sampling techniques were used to select participants from each selected facility, with the first subject chosen at random. The random starting number was used for retrieving maternal records. Each selected health facility shared the required number of clients in Probability proportional to size (PPS) allocation by using the list from the registration books as a sampling frame and then selected the required number of participants by systematic random sampling technique.

### Data collection

The well-structured survey questionnaire, adapted from previous studies (WHO 2001:208), MEASURE DHS project (EPHI & ICF 2021:207; WHO 2015:97) was translated into local and English versions by language experts to maintain consistency in ideas. The data were collected by 25 data collectors, including Obstetrics and Gynecology residents and Senior Midwives working for the recruited health facilities and supervised by a midwifery background public health expert.

### Data analysis

The study used SPSS version 26 for data analysis, with descriptive analysis producing tables and cross-tabulations for complex data. Mean or median were used to describe variables. The Shapiro–Wilk test was used to evaluate data normality. Linear regression was used to determine mean change in dependent variables.

### Validity and reliability

To ensure the accuracy of the results, the authors made modifications to a questionnaire that had been used before and used it in this investigation (WHO 2001:208). The questionnaire was originally developed by the MEASURE DHS project (EPHI & ICF 2021:207; WHO 2015:97). The pilot study was conducted to ensure the reliability of the data collection instrument and determine what relevant data could be obtained from the participants. This was accomplished by using an interview guide and questionnaire, and consistently obtaining the same answers from the participants’ responses over time.

### Ethical considerations

Both the Ethiopian Public Health Institute (protocol number EPHI-IRB-527-2023) and the University of South Africa’s College of Human Sciences (ethics clearance reference number: 10347143_CREC_CHS_2023) have granted ethical approval for the study. Upon obtaining ethical permission, all study activities were conducted in accordance with established research standards. The study adhered to three ethical principles: beneficence, justice and respect for persons. Permission letters were obtained from the city administration health bureau, hospitals and health centres. The questionnaire was designed to ensure participant anonymity and the privacy of their information during data collection. Furthermore, additional security measures were implemented to protect participant information, as the data were gathered using Open Data Kits (ODKs).

## Results

### Socio-demographic characteristics of exit-interview participants

The study involved 354 women who received ANC, delivery care and PNC, with a 100% response rate. As indicated in Table 1, the majority (85.9%) of the respondents’ age were between 20 years and 34 years. Just 11.3% of the population was over 40 years old, and the remaining 2.8%, were in the 15–19-year age range. The exit-interview respondents were divided into pregnant, woman who gave birth and postpartum women. The respondents were urban dwellers, residing in the capital city of the country. First-time mothers constituted 25.1% of the sample, while 45.8% had one to three childbirths. More than half (55.1%) of participants had a diploma or higher, while 7% had no formal education. The study revealed that 76.7% of respondents were Orthodox Tawhido Christians, 75.1% were married and 63.3% spent less than 30 min to reach health facilities. Women’s wealth distribution varied, with 12.7% falling into the lowest quartile and 30.8% falling into the highest (Table 1).

**TABLE 1:** Socio-demographic characteristics of exit-interview participants to assess maternal healthcare services in public health facilities of Addis Ababa, Ethiopia, January 2024 (*N* = 1062).

Variables	Category	Frequency	Percentage
Age (years)	15–19	30	3.0
	20–34	912	86.0
	35–49	120	11.0
Total	-	1062	100.0
Educational background	No formal education	72	7.0
	Elementary, junior or high school	405	39.0
	Diploma and above	585	55.0
Total	-	1062	100.0
Marital status	Single (unmarried)	93	9.0
	Married	798	75.0
	Divorced	114	11.0
	Widowed	192	18.0
	Separated	-	-
Total	-	1062	100.0
Number of children	No children	267	25.0
	1–3	486	46.0
	> 3	309	29.0
Total	-	1062	100.0
Monthly household income (ETB)	≤ 3000	453	13.0
	3001–6000	330	31.0
	6001–9000	270	25.0
	> 9000	327	31.0
Total	-	1062	100.0

### Utilisation of maternal healthcare services

This study analysed maternal health service utilisation indicators, revealing a 70.8% overall utilisation of services, with ANC at 85.5%, delivery at 71.58% and PNC at 55.4%. In the health facilities, there was a noticeable variation in the use of all three services (ANC, institutional delivery and PNC). The utilisation of all three services was comparatively high at the public health centres. Women who had at least four ANC visits from public health centres constituted more than two-thirds (67.4%) of the sample. Of the women who gave birth in health facilities, 72% gave birth at public health centres. The majority of women who utilised PNC services did so from public hospitals. Of the women who attended ANC, 91% gave birth in the health facilities.

### Utilisation of maternal healthcare services among pregnant women

Using the three ANC service adequacy indicators – the timing of the first visit, the frequency of visits and ANC service items – this study evaluated the extent and determinants of sufficient prenatal care service utilisation among the mothers in public health facilities of Addis Ababa. Acknowledging pregnancy concerns and managing birth issues are closely related to the number and timing of antenatal visits and the prescribed service items obtained during antenatal appointments.

The types of maternal healthcare services that clients used, the number of ANC visits and the proportion of clients who experienced pregnancy-related problems while undergoing care in Addis Ababa’s public health facilities are all shown in Table 2. Of the 354 pregnant mothers who attended ANC visits, 272 (76.8%) had four or more visits. Almost all (99.7%) women who sought maternal health services had a physical examination, and 92.9% received an ultrasound checkup. Nearly one-third, 110 (31.1%) of pregnant women who visited health facilities were found to have pregnancy-related complications, and 104 (94.6%) of them were referred to another hospital for further treatment (Table 2).

**TABLE 2:** Utilisation of maternal health care services among pregnant women who visited public health facilities in Addis Ababa, Ethiopia, January 2024 (*N* = 354).

Variables	Category	Frequency	Percentage
Did you receive medical care during your pregnancy at the public health facility?	Yes	354	100.0
	No	0	0.0
Total	-	354	354.0
How many times did you visit the clinic during your pregnancy?	1 to 3 visits	82	23.0
	More than 3 visits	272	77.0
Total	-	354	100.0
What health services did you receive when you visited the clinic during your pregnancy? (multiple responses)	Physical examination	353	100.0
	Gynaecological examination	270	76.0
	Ultrasound	329	93.0
	HIV/STD testing	325	92.0
	Blood tests	320	90.0
	Nutritional supplements	236	67.0
	Tetanus vaccine	328	93.0
Average of the availability of the listed services		-	87.0
Were any complications detected during your pregnancy	Yes	110	31.0
	No	244	70.0
	Total	354	100.0
Were you referred to another hospital for treatment of these complications?	Yes	104	95.0
	No	6	5.0
Total	-	110	100.0

Note: Overall utilisation of ANC services = 85.0.

ANC, antenatal care.

### Maternal healthcare services utilised by women who have given birth

A total of 354 women who attended ANC all received medical care from skilled providers, according to the findings of the exit interview with maternity care consumers. Midwives attended to more than two-thirds (68.1%) of the deliveries; doctors and nurses assisted with the remaining 28.8% and 3.1% of births, respectively. Just about 8 out of 10 (82.5%) respondents reported that they were completely satisfied with the care they received from the skilled birth attendants. Table 3 shows that, of the 62 women who expressed dissatisfaction with their care, 30.6% cited uncertainty of professionals’ skill, 24.2% noticed lengthy waiting time and 16.1% revealed unanticipated negative outcomes for women or newborns. Moreover, 14.5% cited disregard of professionalism by health personnel, 12.9% mentioned the high ultrasound costs and 11.3% cited a lack of infrastructure as reasons for their discontent (Table 3).

**TABLE 3:** Utilisation of delivery care services among women attending antenatal care at the public health facilities of Addis Ababa, Ethiopia, January 2024 (*N* = 354).

Variables	Category	Frequency	Percentage
During delivery, were you attended by a skilled birth attendant (doctor, nurse or midwife)?	Yes	337	95.0
	No	27	5.0
Total	-	354	100.0
Who were you attended by?	Doctor	102	29.0
	Nurse	11	3.0
	Midwife	241	68.0
Total	-	354	100.0
How satisfied were you with the care you received from the skilled birth attendant?	Completely satisfied	292	82.0
	Partially satisfied	50	14.0
	Neither satisfied nor dissatisfied	3	1.0
	Dissatisfied	9	2.0
Total	-	354	100.0
What were the reasons for your dissatisfaction?	Uncertainty on professionals’ skill	19	31.0
	Waiting time	15	24.0
	Bad outcome(s) on baby and/or woman	10	16.0
	Disrespect professionals’ approach	9	14.0
	Cost for Ultrasound	8	13.0
	Lack of infrastructures	7	11.0
	Discomfort with the waiting area	6	10.0
	Leading to unwanted Cesarean Section	6	10.0
	From the 62 dissatisfied women	-	-
Did you experience any complications during delivery?	Yes	65	18.0
	No	289	82.0
Total	-	354	100.0
Did the primary clinic provide emergency care for these complications?	Yes	55	84.0
	No	10	14.0
Total	-	65	100.0
Were you taken to a secondary hospital for emergency care?	Yes	6	9.0
	No	59	91.0
Total	-	65	100.0

Note: Overall utilisation of delivery services -71.0.

### Utilisation of postpartum maternal healthcare services by women

As shown in Table 4, of the 354 women who gave birth in the health facilities, 311 (87.9%) received follow-up care. The overall utilisation rate of PNC among women who gave birth was 55.4%. Eighteen percent (18%) of the PNC-received women made more than two visits to the health facility following birth, compared to 82% who made one or two. The majority (95.5%) of the women who used services had a physical examination, 62.1% used contraceptives and just 29 (9.3%) had a blood test for anaemia (Table 4).

**TABLE 4:** Utilisation of family planning services services among women who gave birth in public health facilities of Addis Ababa, Ethiopia, January 2024 (*N* = 354).

Variables	Category	Frequency	Percentage
Do you receive medical care after delivery?	Yes	311	88.0
	No	43	12.0
Total	-	354	100.0
How many times did you visit the health facility after delivery?	One to two visits	254	82.0
	More than two visits	57	18.0
Total	-	311	100.0
What health services did you receive when you visited the clinic after your delivery? (multiple responses)	Physical examination	297	95.0
	Counselling on breastfeeding	203	65.0
	Contraceptives	193	62.0
	Blood test for anaemia	29	9.0
	Nutritional supplements	143	46.0
	On warning signs of problems	254	82.0
Total	-		60.0

Note: The average for every 311 women who received medical care after delivery = 55.0.

### Factors affecting the utilisation of maternal healthcare services

The findings of a linear regression model’s analysis of the statistical significance of the association between the utilisation of maternal health services and the majority of predictor factors are displayed in Table 5.

**TABLE 5:** Factors affecting utilisation of maternal healthcare services among public health facilities of Addis Ababa, Ethiopia, January 2024.

Variables	*n*	%	*p*	AOR	95% CI
**Travel time to go to the public health facility**					
Less than 30 min	672	63.0	0.048	**2.29**	**1.16–7.35**
30 min to 1 h	255	24.0	-	7.21	0.19–10.52
1 to 1½ h	283	8.0	-	4.17	0.94–52.17
1½ to 2 h	45	4.0	-	15.02	0.33–21.76
More than 2 h	6	0.6	-	1.00	1
**Average waiting time to see medical staff**					
Less than 30 min	612	58.0	0.013	**23.64**	**4.11–46.13**
30 min to 1 h	288	27.0	-	0.92	0.39–5.13
1 to 1½ h	111	10.0	-	4.58	0.14–7.60
1½ to 2 h	36	3.0	-	12.92	0.15–32.72
More than 2 h	15	1.0	-	1.00	1
**Number of ANC visits**					
1 to 3 visits	82	23.0	-	1.00	1
More than 3 visits	272	77.0	0.001	**4.86**	**4.11–12.83**
**Type of services received during pregnancy**					
Physical examination	297	95.0	0.023	**21.09**	**6.15–38.53**
Counselling on breastfeeding	203	65.0	-	11.92	0.75–44.81
Contraceptives	193	62.0	-	9.31	0.43–25.47
Blood test for anaemia	29	9.0	-	0.79	6.29–12.25
Nutritional supplements	143	46.0	-	31.97	0.12–53.68
On warning signs of problems	254	82.0	-	1.00	1
**Facing pregnancy complications**					
Yes	110	31.0	0.005	**13.89**	**2.65–25.18**
No	244	69.0	-	1.00	1
**Level of satisfaction with received care**					
Completely satisfied	292	82.0	<0.05	**4.50**	**2.57–12.09**
Partially satisfied	50	14.0	-	1.48	0.073–6.27
Neither satisfied nor dissatisfied	3	1.0	-	5.02	0.46–31.65
Dissatisfied	9	2.0	-	1.00	1

Note: The bold values are only to indicate the significant association between explanatory variables and the outcome variable which can be adjusted to make them similar to others. AOR, adjusted odds ratio; 95% CI, 95% confidence interval; ANC, antenatal care.

It was found that the duration of the journey to the public health facility significantly affected the utilisation of maternal healthcare. Pregnant women who travelled for less than 30 min used maternal healthcare 2.29 times as much as those who travelled for more than 2 h, according to the data (AOR = 2.29; 95%CI = 1.16–7.35).

The utilisation of maternal healthcare services is significantly influenced by the average client wait time, with those receiving care in less than half an hour having a 23.6-fold higher utilisation rate (AOR = 23.64; 95%CI = 4.11–46.13) than those who waited more than 2 h.

Pregnant women with four or more prenatal care visits are 4.86 times more likely to use maternity care services as compared to women who went no more than three times (AOR = 4.86; 95%CI = 4.11–12.83). Similarly, the utilisation of maternal health services was significantly associated with the physical examination services obtained during pregnancy, the presence of pregnancy difficulties and the patient’s degree of satisfaction with the care they received (Table 5).

The perception and desire of clients to receive the service have increased with the start of electronic records (ERs) registration for ANC and PNC. The majority of clients are content with their electronic maternal records. They get reminders for routine checkups, brief wait times, targeted visits, assistance from knowledgeable staff, courteous services, timely and consistent notifications, a well-equipped facility, on-site medication availability and information on risk reduction techniques (Addis Ababa Health Bureau annual report 2022/2023).

## Discussion

### Utilisation of maternal healthcare services

The study evaluated the utilisation of maternal health services using three indicators: prenatal care services throughout pregnancy, delivery care services during institutional delivery and PNC services within 42 days after delivery. The timing of the first visit, the number of visits and the ANC service items are the three ANC service adequacy indicators that were employed in this study to assess the extent and factors influencing women’s utilisation of adequate prenatal care services in Addis Ababa’s public health facilities, focussing on identifying pregnancy hazards and managing delivery problems. As shown by these parameters, the overall utilisation of maternal health services was 70.8% (85.5% for ANC, 71.58% for delivery and 55.4% for PNC).

The overall utilisation of ANC services was 85.5%, which is higher than that of the studies carried out in Southern Ethiopia, which found that just 23.13% of the pregnant women utilised the available ANC services (Gebreselassie, Id & Wube 2021:1–10), Hawassa University (69.1%) (Shudura, Yoseph & Tamiso 2020:1) and Haryana, India (58.3%) (Chowdhury & Chakraborty 2021:2879). The observed differences in the extent of ANC utilisation across these studies could be attributed to variations in the study period and population, where sociocultural backgrounds and the opinions of impoverished communities regarding ANC use are evident and could impact ANC uptake.

Four antenatal visits are necessary in a focussed ANC programme in order to provide a mother and her infant with the necessary interventions and ensure their safety (Biadgo et al. 2021:1–10; Defar et al. 2020:1–9). Hence 86% of pregnant women who attended ANC visits had four or more visits which is higher than reported in Lucknow, India (37.8%) (Singh Diipti 2019:1–3) and Hawassa University (37.8%). A hospital referral was made for additional care for 94% of the pregnant mothers experiencing pregnancy-related problems. The research’s differing temporal locations and the potential variations in safe parenting practices between countries (Singh et al. 2019) and across Ethiopia’s regions (Gebreselassie et al. 2021) could be the cause of these discrepancies.

The utilisation of delivery care in this study is 71.5%, which is less than a study conducted in Southwest Ethiopia, 86.4% (Yoseph et al. 2020:4), but higher than the study findings of Dessie City, Northeast Ethiopia, which are 43.4% (Yalew et al. 2022:1). The results of this study indicate a coverage of 55.4% for PNC services. This percentage is somewhat higher than the pooled magnitude of PNC utilisation in sub-Saharan African nations (52.48%) (Tessema et al. 2020:5), but lower than the findings of another study conducted in Debretabor town, which reported a coverage of 57.5% (Wudineh et al. 2018:2). These disparities may arise from the fact that women who were well-informed had higher chances of being well-aware, having higher levels of knowledge and expertise in maternal health care, and having more access to these services. The introduction of ER registration for ANC and PNC might help women understand the importance of continued health professional visits for optimal care and safety, and to increase client satisfaction with their services.

Postpartum family planning services were used by 62.1% of the participants in this study. In relation to other research, this result is higher than the findings of studies conducted in Oromia region, 40.7% (Seifu, Yilma & Daba 2020:170), North Shoa zone, 21.3% (Silesh et al. 2022:4), rural Eastern Ethiopia, 18.4% (Mulatu et al. 2020:1) and Arba Minch town, 44% (Wassihun et al. 2021), but lower than the findings in Addis Ababa, 71.8% (Tafa & Worku 2021:6). The increased use of family planning in this finding could have several causes. A residential factor (living in an urban area) with better access compared to regions (CSA 2017:34) could be one of these, leading to a greater awareness among clients about their freedom and responsibility to choose the number, spacing and timing of their children; to improve the health of women and children by lowering the risks of unintended pregnancy, unsafe abortion, maternal and infant mortality; to engage in responsible sexual behaviour; and to reap socioeconomic benefits. It is possible that the different timeframes at which the investigations were conducted contributed to the difference from the earlier Addis Ababa findings.

The study’s findings showed that, in certain public health institutions, the utilisation of ANC, delivery, PNC and postpartum family planning has consistently increased. Several variables have been identified as the cause of these increases, such as the expansion of services, the recruitment of additional healthcare professionals, the provision of round-the-clock emergency maternal healthcare and the provision of 24-h emergency care for mothers, and the division of work within the health facilities, as evidenced from the Addis Ababa Health Bureau report (Addis Ababa Health Bureau annual report 2022/2023).

Moreover, the implementation of ER in public hospitals and FANC has resulted in improvements to the acceptance, accessibility and utilisation of maternal health services, particularly ANC. The ANC registration feature and attendance at these hospitals had consistently increased when the ER and FANC technique was reemphasised. The records that are now available indicate a precise percentage rise per year for the last 2 years. As ER registration for ANC through PNC began, clients’ perceptions of and desires for the service have grown. Most women are happy with their ER mother record, routine checkup reminders, short wait times, focussed visits, professional staff support, polite services, prompt and reliable notifications, a clean and well-equipped facility, access to on-site medication and education about risk reduction strategies.

Women in ANC and delivery in health facilities were encouraged to use PNC after giving birth, following FMoH National Guidelines for Family Planning Service. Many made appointments for PNC within a week, aligning with guidelines (FMoH Ethiopia 2020:1–74). Postpartum women, especially those with complications, prefer returning to the same health facility or advanced facilities, citing improved instruction and counselling during ANC.

To reduce the dangers of pregnancy, women want to know about the ANC phase, have access to routine exams and have skilled attendants to do prenatal checkups and births, based on the findings. Despite the inadequate quality of care they receive, mothers keep visiting the health institution to receive FP, ANC and PNC care. Most clients did not refuse to use maternal health services, except for dissatisfaction with the standard of care. However, as the respondents reply, the majority of women would prefer to speak with a hospital attendant rather than the health centre’s staff members because they lack trust in their abilities. The decisions of a client about where and how to receive maternal health services seem to be influenced by their family, place of residence and marital status.

In comparison, the public health centres had quite high usage of all three services. Approximately two-thirds (67.4%) of the users were women who had received at least four ANC visits from public health centres. Nearly 72% of the women who gave birth in health facilities during this time did so at public health centres. On the other hand, public hospitals provided PNC services to the great majority of women who used them. Ninety-nine per cent of the women who attended ANC gave delivery in health facilities. One possible explanation for this could be the proximity of health centres to clients’ residences, reduced waiting times and low patient load.

According to this survey, public health facilities in Ethiopia provide official free maternity and neonatal health services; however, due to frequent shortages of drugs and supplies, clients were sometimes requested to procure missing items. Certainly, providing free maternity health care improves utilisation and accessibility (Nuamah et al. 2019:7). Nonetheless, the lack of knowledge among women about free maternal health services, particularly in underprivileged populations, has a negative effect on the use of health care.

This study also revealed the presence of clients’ negative opinions regarding public health facilities, particularly maternity services. They did not even want to go to the hospitals because of the poor quality of certain facilities and services they received. The attitudes of the skilled attendants, the facilities, the students and other elements all contribute to their lack of interest in public health institutions.

### Factors affecting utilisation of maternal healthcare services

Using a linear regression model, the statistical significance of the relationship between the utilisation of maternal health services and the most predictor factors was examined. It was discovered that the length of time spent travelling to the public health facility had a substantial impact on the utilisation of services related to maternal health.

The result (AOR = 2.29; 95% CI = 1.16–7.35) indicates that pregnant women who travelled for less than 30 min utilised maternal health services 2.29 times as much as those who went to the facility for more than 2 h. This study’s findings are consistent with those of a study carried out in Ghana by Nuamah et al. (2019:7). Similar conclusions were also made in rural southern Ghana by (Manyeh et al. 2020:5). In Mogadishu, Somalia Ahmed and Husein (2020:1649) found that the utilisation of maternal health services was enhanced by shorter travel distances to health facilities. However, some research conducted in the globe (Galle et al. 2023:6), in sub-Saharan Africa (Modisakeng et al. 2020:6) and in Arba Minch (Wassihun et al. 2021:5) did not identify any significant association between maternal health services and travel times to health facilities.

The utilisation rate was 23.6 times higher for pregnant women who received care in less than 30 min waiting hours (AOR = 23.64; 95%CI = 4.11–46.13) compared to those who waited longer than 2 h. This is consistent with research from another study carried out in Ethiopia (Bradley et al. 2021:226), India (Lefevre et al. 2019:11) and sub-Saharan Africa (Wigley et al. 2020:7; Kolleh et al. 2022). Women’s waiting times were shortened, which led to a rise in the use of maternal health services, especially for women living in places where roads, geography and distance make it difficult to go to health facilities in a timely manner. However, the findings of studies conducted in Africa (Kolleh et al. 2022:7; Frank 2020:696) did not show any association. Nonetheless, research suggests that receiving care in private health facilities can lead to favourable outcomes, including reduced wait times and more provider time dedicated to mothers (Biadgo et al. 2021:8–10).

Compared to women who went less frequently or no more than three times, pregnant women who had four or more prenatal care visits were 4.86 times more likely to utilise maternity care services (AOR = 4.86; 95%CI = 4.11–12.83). Similarly, the utilisation of maternal health services was significantly correlated with the physical examination services obtained during pregnancy, the presence of pregnancy difficulties and the patient’s degree of satisfaction with the care they received.

## Strengths and limitations

### Strengths

This study used various techniques for data collection, including ODK. Open Data Kit significantly enhances a study by providing robust data collection tools that work offline, ensuring data accuracy with built-in validation rules, and enabling real-time data analysis with its instant upload features once a connection is available. Using an instrument or data collection tool that has been previously validated could potentially be seen as a strength of this study. Also, because women participated in the study, it was possible to identify shared perspectives on the use of services, experiences and difficulties.

### Limitations

Some limitations to the study are evident because not all components of basic maternal care services were assessed. In addition, the results might not apply to the entirety of Ethiopia because the study was limited to Addis Ababa.

## Recommendations

Policymakers, health managers, healthcare institutions and healthcare providers should all play their specific roles in order to improve the utilisation of maternal health services. Any developed strategies should be tailored to both service users and providers. In addition to establishing functional links between facilities and health systems, health managers should guarantee the integration, quality and accessibility of service-delivery packages. They also need to promote the application of evidence-based practices for PNC, ANC and intrapartum care. To improve maternal health services utilisation, health facilities should protect women’s privacy, empower women, provide adequate medications and supplies, and enhance care continuity. Healthcare providers should also recognise pregnancy-related hazards and regularly monitor service provision and adherence to standards. Women should also be encouraged to take charge of their own healthcare, often with assistance from their families. Pregnancy-related risks, health behaviours, medications, preventive care and information should all be offered.

## Conclusion

The study examined how mothers’ use of perinatal care in Addis Ababa’s public health facilities was impacted by service adequacy factors. It was shown that access to and utilisation of various maternal health services are frequently influenced by financial conditions, which results in unequal service provision at some health care facilities and providers. In comparison to delivery and PNC, ANC services are used more frequently. As the city is the nation’s capital and the location of the majority of the nation’s resources, even if the service utilisation finding appears to be greater than comparable findings from the regions, it is yet below ideal and expected levels.
